# A Systematic Review of Associations and Predictors for Job Satisfaction and Work Engagement in Prehospital Emergency Medical Services—Challenges for the Future

**DOI:** 10.3390/ijerph20054578

**Published:** 2023-03-04

**Authors:** Beatrice Thielmann, Robin Schwarze, Irina Böckelmann

**Affiliations:** Institute of Occupational Medicine, Medical Faculty, Otto-von-Guericke-University, Leipziger Straße 44, 39120 Magdeburg, Germany

**Keywords:** paramedic, ambulance service, workload, stress, job performance, strengthening the future

## Abstract

Ambulance services are changing, and the SARS-CoV-2 pandemic has been a major challenge in the past three years. Job satisfaction and work engagement are important characteristics for a healthy organization and success in one’s profession. The purpose of the current systematic review was to evaluate the predictors of job satisfaction and work engagement in prehospital emergency medical service personnel. Electronic databases, such as PubMed, Ovid Medline, Cochrane Library, Scopus, Web of Science, PsycINFO, PSYNDEX, and Embase, were utilized in this review. Predictors (ß coefficient, odds ratio, rho) of higher job satisfaction and work engagement were examined. Only prehospital emergency medical service personnel were considered. The review included 10 studies worldwide with 8358 prehospital emergency medical service personnel (24.9% female). The main predictor for job satisfaction was supervisors’ support. Other predictors were younger or middle age and work experience. Emotional exhaustion and depersonalization, as burnout dimensions, were negative predictors for higher job satisfaction or work engagement. Quality demands for health care systems are a significant challenge for future emergency medical services. The psychological and physical strengthening of employees is necessary and includes constant supervision of managers or facilitators.

## 1. Introduction

Working in (prehospital) emergency medical service (EMS, ambulance service) is volatile, uncertain, complex, and ambiguous (VUCA) [1]. Volatile refers to the fluctuations and fast-moving nature that we experience every day in the modern, digitalized world. Uncertain refers to the aggravated unpredictability of events. Complex means that many influencing factors are interdependent and interact with each other. Ambiguity means more difficult orientation. Simple and straightforward attributions are rare [1]. 

Regarding ambulance services, emergency scenes and situations, patients, and relations are constantly changing and cannot be influenced by emergency medical services [1]. The treatment of patients is far from the controlled circumstances of classic standardized production processes [1]. The SARS-CoV-2 pandemic is a major challenge for a VUCA workplace [2], including the prehospital EMS [3]. The subjectively experienced stress and recovery between the first and second waves of the SARS-CoV-2 pandemic have negatively affected emergency medical service personnel (EMSP) [4,5]. In addition, the population’s demand for quality in medical care systems is increasing and constitutes a major challenge [6]. Growing gaps in the area of family doctor systems, an increase in (older) patients seeking help (often in rural areas), and rising cost-performance pressure are influencing factors for a reorganization of modern emergency medicine and thus present new challenges [6]. 

Stress is an interaction between an individual and his or her environment according to the subjective perception and assessment of stressors [7]. Thus, one basic theoretical model of the development of health impairments as a consequence of occupational stress situations is the job demand control model introduced by Karasek in 1979 [8]. In this model, strain and stress factors in the work environment are evaluated, promoting health in the workplace. Job demands and decision latitude for action contrast with each other in this context. The model was extended to the job demand control support model by Johnson and Hall in 1988 [9]. A lack of social support can further decrease mental health and well-being [9,10]. A hypothesized model of work-related well-being demonstrated a multidimensional construct and included the factors job satisfaction, occupational stress and work engagement [11]. Job satisfaction is defined as the employee’s evaluation of the work context [12]. It is the employee’s overall feeling toward the workplace and its facets [13] and includes, for example, colleagues, promotion, and pay [11]. Schaufeli et al. (2002) proposed the theoretical construct of work engagement, which describes a fulfilling and positive work attitude. In fact, it can be attributed to the fact that some employees find joy in their work regardless of strenuous work demands and stressors. [14]. It is known that EMSP have particularly high motivation to perform their job [15,16]. Intrinsic motivation is a predictor of job performance [17]. Personal health and mental well-being play critical roles in the emergency medical service (EMS) profession. Employees of prehospital EMS are highly susceptible to occupational burnout [18,19,20] and work-related suicide exposure [21,22]. Characteristics and conditions of the workplace environment are also important [18,19,20,23].

In view of the lack of professionals [24], increasing numbers of operations [25,26], and fluctuations in ambulance service [27], the aim of this systematic review was to examine the predictors of higher job satisfaction and work engagement in prehospital emergency medical service personnel. The impact of the SARS-CoV-2 pandemic on job satisfaction and work engagement should also be considered.

## 2. Materials and Methods

### 2.1. General Procedure

A systematic literature review was designed in accordance with the Preferred Reporting Items for Systematic Reviews and Meta-Analysis (PRISMA) statement for reporting systematic reviews [28,29]. The following electronic databases were included: PubMed, Ovid Medline, Cochrane Library, Scopus, Web of Science, PsycINFO, PSYNDEX, and Embase. We used the major databases accessed by our university (including the possibility of receiving publication). The deadline for literature search was 7 July 2022.

The search terms are listed in Table 1. Medical Subject Headings (MeSH) were used, which are defined search terms of the National Library of Medicine used for indexing articles for PubMed [30]. For example, the MeSH term “ambulances” includes the following headings: health care facilities, manpower, services, health services, emergency medical services, transportation of patients, ambulance diversion, ambulances, air ambulances, and stretchers. The MeSH term “emergency medical technicians” includes, among other definitions, paramedics. These are no longer listed separately.

The full study protocol is downloadable at Prospero: https://www.crd.york.ac.uk/prospero/display_record.php?ID=CRD42022337637, accessed on 6 November 2022. The inclusion and exclusion criteria are shown in Table 2.

Citavi 6 (Swiss Academic Software, Wädenswil, Switzerland) was utilized to import the references from databases to the document reference manager. Duplicates were recognized by Citavi and removed by not transferring. Authors B.T. and R.S. separately screened the titles and abstracts based on the inclusion and exclusion criteria. After summary viewing, the full text of each relevant article was then accessed. Authors B.T. and R.S. again reviewed the full texts of these articles in an independent manner. Differences were discussed with the third expert (I.B.). 

### 2.2. Assessment of Methodological Quality

The Quality Assessment Tool for the Dictionary of Quantitative Studies (EPHPP) [31] and the revised Scottish Intercollegiate Guidelines Network (SIGN) rating system [32] were used to assess the methodological quality of the included studies. The EPHPP tool can be used for study designs such as cohort analytic (two groups pre + post), case-control, cohort (one group pre + post (before and after)), interrupted time series, or other specifications. The EPHPP is based on the categories of selection bias, study design, confounders, blinding, data collection, withdrawals, and dropouts [31]. Each element was rated as either strong, moderate, or weak for each article. Studies with two or more weak categories were classified as weak overall [31]. The Scottish Intercollegiate Guidelines Network (SIGN) uses a revised grading system for recommendation levels of evidence and grades of recommendations [32]. Significant questionnaire variables were assessed from the studies. The results of these articles were noted in Excel Microsoft 365 (Microsoft Corporation, Redmond, WA, USA).

### 2.3. Interpretation of the Statistical Results

The following notations are provided in the text to interpret the significance of the studies: * is *p* < 0.05, ** is *p* < 0.01, *** is *p* < 0.001. In interpreting the Pearson’s or Spearman’s correlation coefficients (rho) according to Akoglu [33], <0.39 means a low effect, 0.40–0.59 means a moderate effect, and >0.6 means a strong effect. The interpretation of the ß coefficient from linear regression analysis is comparable to Pearson’s r: 0.1–0.3 is a low effect, 0.31–0.50 is a moderate effect, >0.5 is a strong effect [34]. In the multifactorial analysis of variance according to Cohen [35], η² can be 0.01 (small effect), 0.06 (medium effect), or 0.14 (large effect). An odds ratio (OR) of greater than 1.00 indicates a predictor of job satisfaction or work engagement [36].

## 3. Results

### 3.1. General Results

**Figure 1:**
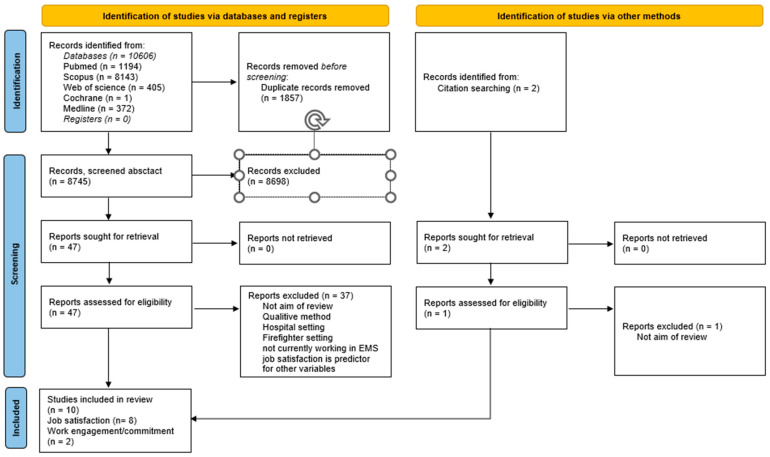
PRISMA flow diagram for systematic reviews.

From the databases, 10,606 citations were imported to Citavi. Two articles were added from a manual search. A total of 8747 references were evaluated. After titles and abstracts were screened for inclusion and exclusion criteria, 47 full texts were evaluated for further acceptability. Of these, 37 articles did not meet the inclusion criteria. Thus, 10 studies were selected for the current review. The flowchart of the review process among PRISMA [29] is shown in Figure 1.

The review differentiates between:

- Studies that used “job satisfaction” questionnaires [15,24,37,38,39,40];
- Studies that used item or subscale “job satisfaction” in other questionnaires or nonstandardized questionnaires [41,42];
- Studies that focused on “work engagement” or “commitment” questionnaires [43,44].

The differentiation is useful because there are mainly validated and standardized questionnaires about job satisfaction, but some studies only use subscales of job satisfaction or do not ask standardized additional questions. Because the authors’ own supplemental questions have not been validated, they are considered non-standardized. We did not intend to exclude these studies.

In all included studies, a total of 8358 prehospital EMSP were surveyed, ranging from 91 [39] to 2590 [24] subjects per study. All studies reported sex. One study examined only males in the prehospital setting [37]. One study investigated 51.4% females [44]. The average representation of females in all other studies was 24.9%. One study did not report age [15]. Four studies provided age groups or a range of ages from 18 to over 50 years [24,37,38,42]. The mean age of the subjects in all other studies was 36.5 ± 10.6 years. The elaboration of the studies showed inconsistent information and no standardized professional classification of professional training of the EMSP. Involved in the studies were paramedics (PM), emergency medical services professionals, emergency medical technicians (EMT), or drivers who worked in prehospital emergency medical services. No study considered emergency physicians. The studies were performed almost worldwide. Four studies were conducted in Europe [24,40,42,44], and three studies were performed in North America [15,39,41,45,46,47]. In addition, one each came from Asia [37], Australia [38], and Africa [43].

### 3.2. Results of Studies That Used “Job Satisfaction” Questionnaires

The results of six included papers [15,24,37,38,39,40] are shown in Table 3. In each of these papers, job satisfaction was evaluated considering other variables, e.g., sex, violence, and support. Unless otherwise noted, a high score also means high job satisfaction.

Basabr et al. (2018) [37] used the 18-item Persian version of the Brayfield–Rothe job satisfaction questionnaire with a cutoff score of 57 points [48,49]. The authors compared prehospital and hospital EMSP in Iran. Only males work in prehospital EMS (authors referred to them as nurses). It seems that there may be no special training such as paramedic training. Control variables were sex, education level, monthly income, and work experience. The EMSP (62.6% of the 115 males) had moderate job satisfaction (45.25 ± 11.29), which was not different from that of the hospital nurses. The authors performed a linear regression analysis and revealed the variables of sex, education level, work experience, and income level as significant moderate determinants of job satisfaction. There was a negative and significant relationship between sex and job satisfaction (ß = −0.202). The job satisfaction of female nurses was lower than that of male nurses, but they worked in different areas (males work in prehospital EMS). These factors explained 20% of the variance (R² = 0.201) of the results.

Brough (2005) [38] used the 15-item Warr, Cook, and Wall scale for measuring job satisfaction [50] of 119 EMSP in Australia. The authors examined the influence of experiences of verbal and physical violence, as described by Brough and Mansell (2001) [51], and social support from supervisors or colleagues using Caplan, Cobb, French, Harrison, and Pinneau’s Social Support Scale (1995) [52]. Demographic variables such as sex, marital status, age, and tenure were examined but were not significantly related to job satisfaction. Verbal violence (r = −0.31 ***) was significantly negatively associated with job satisfaction. Physical violence was not relevant. Supervisor support (r = 0.44 ***) and colleague support (r = 0.33 ***) were positively associated with job satisfaction. A hierarchical multiple regression for job satisfaction showed that verbal violence was a negative predictor of job satisfaction (ß = −0.21 *) but not under the influence of supervisor or colleague support. Both types of support were also predictors of job satisfaction (supervisor ß = 0.28 **, colleagues ß = 0.21 **). The predictor variables accounted for 26% of the variance in job satisfaction. It did not matter whether EMP received high or low levels of supervisor support; they responded similarly to increasing experiences of verbal abuse.

Carrière and Boruque (2008) [39] examined 91 Canadian EMSP. The authors used the short form of the Minnesota Satisfaction Questionnaire (MSQ) developed by Weiss et al. (1967) [53]. Intrinsic and extrinsic job satisfaction were examined with a 7-point Likert scale (from 1, very dissatisfied, to 7, very satisfied). One of the central questions of this study was whether communication satisfaction mediates the relationship between communication practices and job satisfaction. To clarify the question, the authors also used two questionnaires. The first was the Communication Audit Survey (CAS) by Goldhaber and Rogers (1979) [54] and Downs and Ardian (2004) [55] for measuring internal communication practices. The subscales are information received from others, information sent to others, follow-up on information sent to others, key sources of information, timeliness of information received from key sources, organizational communication relationships, organizational outcomes, and channels of information. The second was the Communication Satisfaction Questionnaire (CSQ) developed by Downs and Hazen (1977), with the following dimensions: communication climate, communication with supervisors, organizational integration, effects of organizational communication, media quality, horizontal and informal communication, organizational perspective, personal feedback, and communication with subordinates. [56]. The following demographic variables were used as control variables: age, sex, highest level of education, marital status, number of children, organizational tenure, shift duration, and shift pattern. The mean MSQ score was 4.74 ± 0.97. The control variable “organizational tenure” had a low negative association with job satisfaction (r = −0.32 **). The control variable “shift pattern” (r = 0.30 **) had a weak association, CAS (r = 0.48 **) had a moderate association, and CSQ (r = 0.72 **) had a strong positive association with job satisfaction. In the first step, study variables related to job satisfaction were analyzed (bivariate analysis). The control variables accounted for 28.4% of the variance in the job satisfaction variable, but only “organizational tenure” (ß = −0.31 *) and “shift pattern” (ß = 0.43 **) were significant. Communication practices were positively related to job satisfaction (ß = 0.47 ***) and accounted for an additional 19.8% of the variance in job satisfaction. In the next step, testing for mediation effects involved multiple-stage regression analyses. In summary, the control variables accounted for 20.8% of the variance in the job satisfaction variable, but only “organizational tenure” (ß = −0.29 *) and “shift pattern” (ß = 0.35 **) were significant. Communication satisfaction was positively associated with job satisfaction (ß = 0.66 ***) and accounted for an additional 37.0% of the variance in job satisfaction. The mean score of job satisfaction was 85.32 ± 13.55 (maximum 132 points). There was a moderate positive association between job satisfaction and quality of life (r = 0.508 **). The variables age group, work experience, leisure and self-rated status were significant for quality of life and considered in the multiple regression analysis. These variables explained 31.8% of the variance in quality of life, which was significantly higher for those with higher levels of job satisfaction. Lower self-rated health status was a negative predictor of quality of life. Job satisfaction (ß = 0.399 ***) and leisure were positive predictors of quality of life. Age and work experience were not significant.

Eiche et al. (2021) used the job satisfaction questionnaire by Neuberger and Allerbeck [57] and compared 2500 EMSP with the general population. Each job satisfaction subscale, i.e., “my activity”, “my colleagues”, “my supervisor”, “my development”, “my working conditions”, “organization and management”, and “my payment”, was rated (with 1 = “yes” to 4 = “no”) significantly lower (each subscale with *p* < 0.01) by the paramedics than by the general population. The last two subscales offered the largest differences between the two groups. Binary logistic regression analyses were performed to identify factors that influenced job satisfaction (comparison factors low and high job satisfaction). “Notfallsanitäter in training” (OR 3.085, *p* = 0.03), “Rettungsassistent” (OR 0.691, *p* < 0.01), “0–5 years of service” (OR 1.588, *p* < 0.01), “age 18–30 years” (OR 1.977, *p* < 0.01), and “age 21–40 years” (OR 2.263, *p* < 0.01) were significant factors. It should be noted that “Notfallsanitäter” is the highest nonphysician EMS in Germany.

Stefurak et al. (2020) surveyed 1403 EMP on the basis of the job satisfaction questionnaire by Naff and Crum 1999 (four items) [58] and Vandenabeele 2009 (five items) [59]. The study focused on the concept of public service motivation, which is centered on promoting the common good and is fueled by loyalty and a sense of duty to the public [15]. It was ultimately the most powerful predictor of job satisfaction.

Sterud et al. (2011) [40] used the job satisfaction scale by Warr et al. 1979 [50] in a longitudinal study among 324 EMSP at two time periods (2005 and one-year follow up). The mean scores of job satisfaction at both time periods were equal. The bivariate Pearson’s correlation indicated important significant associations between lower job satisfaction and “emotional exhaustion” at t2 (r = 0.500) *, “psychological distress” at t2 (r = 0.300) *, “lack of leader support” (r = 0.360) *, and “challenging job tasks” (r = 0.30)*. The multiple regression model showed predictors for lower job satisfaction such as “lack of leader support” (ß = 0.32) *** and “challenging job task” (ß = 0.27) **. All analyses were adjusted for T1 levels on the relevant dependent variable (depending on the selected model). 

**Figure 2:**
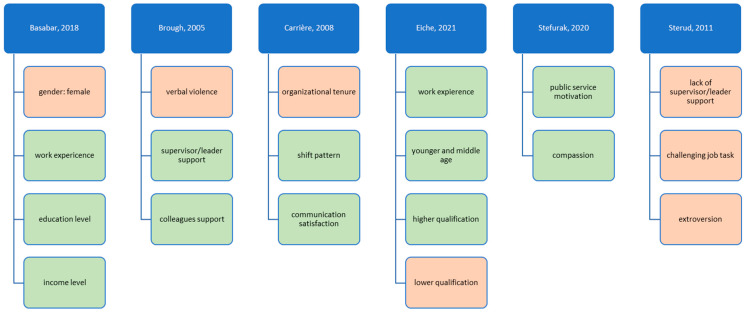
Predictors for (higher) job satisfaction. Notes: Green is positive, red is negative.

In summary, Figure 2 shows an overview of all significant predictors for job satisfaction in reviewed studies that used standardized questionnaires of job satisfaction.

### 3.3. Results of Studies That Used Items for “Job Satisfaction” in Other Questionnaires

The results of the included papers [41] are shown in Table 4. They integrated job satisfaction items into other questionnaires.

Nowrouzi-Kia et al. (2022) [41] investigated career and job satisfaction as subscales in the 23-item Work-related Quality of Life (WRQoL) scale developed by van Laar et al. (2007) [60]. The mean score of job satisfaction was 19.45 ± 3.87 points and was declared lower. Only sex (female) predicted higher job satisfaction.

Roth et al. (2021) [42] surveyed EMSP on the basis of a questionnaire from the RN4CAST projects (nurse forecasting in Europe) [61]. The study showed a strong negative correlation between the job satisfaction variable “satisfaction with current workplace” and the burnout dimension “emotional exhaustion” (r = −0.373) **. The regression analysis demonstrated that burnout variables “emotional exhaustion” and “depersonalization” were significant negative predictors for the following variables of job satisfaction: current workplace, choice of profession, and professional status.

**Figure 3:**
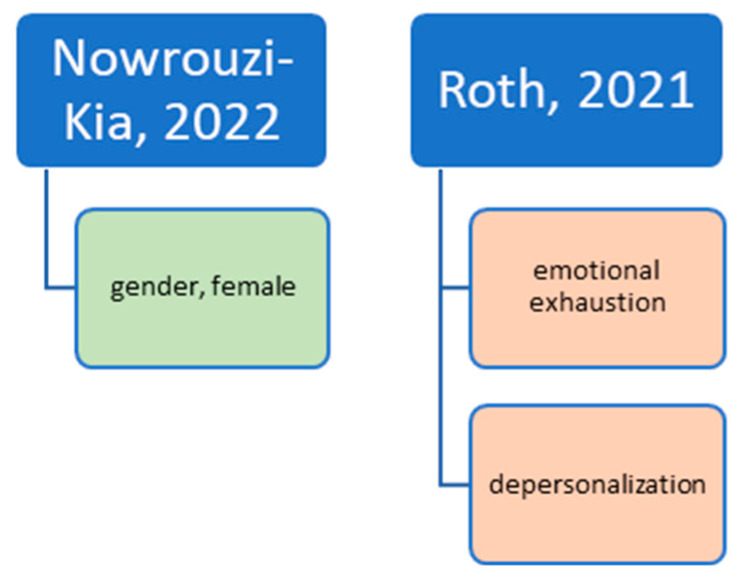
Predictors for (higher) job satisfaction from studies using job satisfaction items integrated in other questionnaires. Notes: Green is positive, red is negative; *only correlation, showed trend.

In summary, Figure 3 shows an overview of all significant predictors for job satisfaction in studies that used job satisfaction items in other questionnaires.

### 3.4. Results of Studies That Investigated Work Engagement/Commitment

Two papers investigated predictors for work engagement or commitment [43,44]. The results are presented in Table 5.

Naudé and Rothmann (2006) [43] used the Utrecht Work Engagement Scale introduced by Schaufeli et al. (2002) [14] for the evaluation of work engagement. Emotional exhaustion (r = −0.280) ** and depersonalization (r = −0.320) ** as dimensions of burnout were significantly negatively associated with work engagement. Personal accomplishment (as a burnout variable) was positively associated with work engagement (r = 0.061) **. Sense of coherence was also positively associated with work engagement (r = 0.410) *. A multiple regression analysis showed that sense of coherence was the only statistically significant predictor for work engagement (ß = 0.016) **.

Setti et al. (2018) [44] used the five-item affective commitment scale of the Organizational Commitment Questionnaire [62]. Affective commitment was positively related to supervisors’ support (r = 0.280) ** and colleagues’ support (r = 0.230) **. Role conflict directly influenced affective commitment (ß = 0.16). Additionally, supervisors’ support directly increased affective commitment (ß = 0.250), and job burnout decreased affective commitment (ß = −0.220).

**Figure 4:**
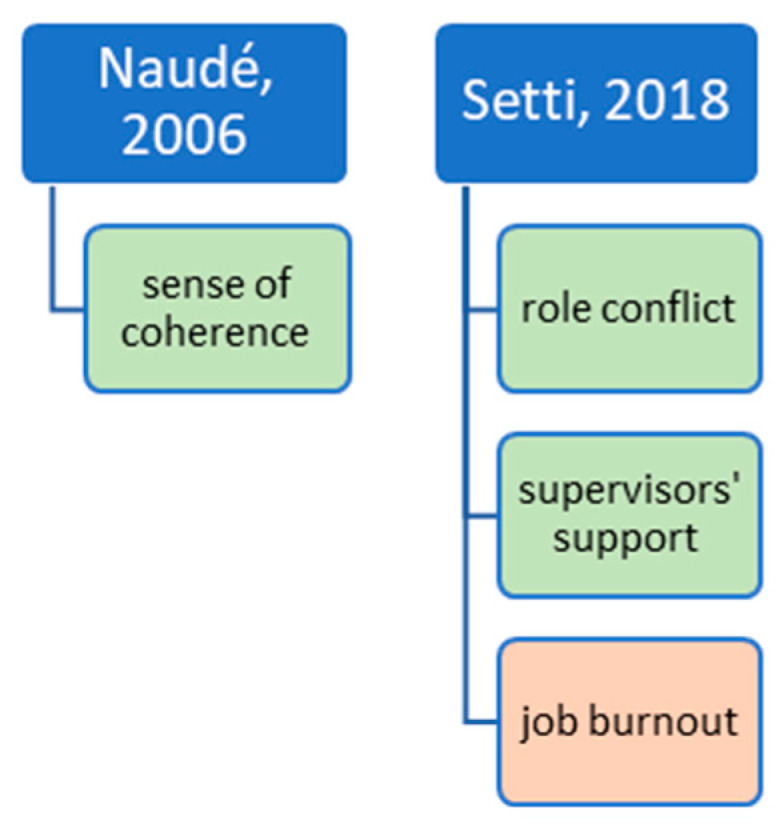
Predictors for work engagement or commitment.

In summary, Figure 4 shows an overview of all significant predictors for job satisfaction in studies that used job satisfaction items in other questionnaires.

### 3.5. Quality Assessment of Included Studies

Considering the components of the rating, i.e., selection bias, study design, confounders, blinding, data collection methods (strongest component in all studies), withdrawals and dropouts (not applicable), intervention integrity, and analysis appropriate to question, all studies were rated weak according to the quality assessment tool for the quantitative studies dictionary by the EPHPP [31].

The second tool, developed by the Scottish Intercollegiate Guidelines Network (SIGN) [32], was evaluated. A level of evidence of 2 (e.g., case-control or cohort studies with a high risk of confounding, bias, or chance and a significant risk of a noncausal relationship) was obtained.

## 4. Discussion

Although emergency service personnel seem to be motivated by high internal factors and are action-oriented and highly committed to their work [16], new aspects of a changing work environment were found to reduce job satisfaction and work engagement and were associated with job and career changes [27]. This systematic review examined predictors for higher job satisfaction and work engagement among prehospital emergency medical service employees. Ten studies worldwide were considered in this review. No study considered emergency physicians. The studies were evaluated according to the tools introduced by EPHPP and SIGN, which are quality assessment tools for quantitative studies. Using these tools, the studies were classified as weak or as having low evidence. All studies examined different predictors of job satisfaction and work engagement, so evaluability is limited. Nevertheless, the studies show a directional trend to keep job satisfaction and work engagement high. No conclusions can be made about the impact of the SARS-CoV-2 pandemic on job satisfaction and work engagement because no studies conducted before and after comparisons.

Studies have demonstrated that leadership behavior and support play a major role in the awareness of stress and job satisfaction [63,64,65]. This review confirmed these claims. Supervisors’ support was the greatest predictor of job satisfaction and work engagement [38,40,44]. Experienced support from colleagues was also a positive predictor of job satisfaction [38], which was confirmed in other studies [66,67]. Support also included, for example, practical skill exercises, theoretical knowledge, experience-based knowledge, theoretical support, management, and organizational support [65].

Another review showed elevated levels of burnout risk and a moderate negative correlation between job satisfaction and MBI emotional exhaustion and depersonalization [68]. Both burnout dimensions were negative predictors for job satisfaction and work engagement in the current review [42,44]. Significant statistical differences regarding the mean burnout in the different groups of educational levels and qualification levels were shown [69]. Sociodemographic data were also predictors of job satisfaction and work engagement. Being of younger or middle age was a predictor for job satisfaction in the current review [24]. In the study by von Okada et al. (2005), mental stress was reported more frequently by older paramedics [70]. Another study demonstrated that as age increased, resilience was lower, and those with lower resilience scores were less satisfied with their work [71]. This is countered by professional experience, which was a positive predictor of job satisfaction in the current review [24,37].

The percentage of females in the ambulance service is increasing. The number of studies on the stresses and strains on females in the ambulance service can generally be classified as low. Previous studies usually considered the ambulance service as a whole or considered females as mirror images of male emergency personnel, since the studies of previous samples of women often included very low numbers of participants. The average proportion of females is approximately 25%. The current review found a negative and significant relationship between sex and job satisfaction, but the comparison was between males in the prehospital EMS setting and females in the hospital setting [37]. Therefore, this interpretation should be made cautiously. Another study demonstrated that females reported higher job satisfaction [41]. Further research is necessary in this regard. The trend shows that ambulance services can no longer be considered a typical male profession in the future. A typical male profession exists when more than 70% of the employees are men or vice versa for a typical female profession [72]. This can also vary from state to state and is typical in Germany [73].

### 4.1. Challenges in Ambulance Services

Due to work environment changes, new challenges have arisen in ambulance services. It should be clarified that job satisfaction or work engagement is one of the effective variables of organizational performance and supports a significant negative relationship with turnover intention [74] but is affected by a variety of variables, which also change with respect to time, place, and social conditions [37]. The current SARS-CoV-2 pandemic is an extreme challenge for more than prehospital emergency medical services [3]. Job satisfaction and work engagement can be maintained if employees have the qualifications, motivation, and health to cope with their stresses and strains [6]. This involves a “personal responsibility” on the part of each EMSP. The company has a duty to provide necessary resources, referred to as “corporate responsibility” [6].

For larger agile teams among the VUCA workplace, such as those of ambulance services, it is helpful to employ an experienced moderator to support the work process with the help of suitable methods [1]. In addition, there are employee-related aspects of ensuring competence, awareness of one’s own contribution, and communication that should be trained [1]. Considering the high burnout and suicide rates from the literature, suicide prevention, stress management skills, and ongoing education in suicide intervention are needed [75].

The need for better pay, benefits, and career advancement opportunities as reasons for leaving the emergency medical service [27] should be common themes for discussion in the future. There is a great need for future sex-specific research in both qualitative and quantitative research because the percentage of females in ambulance service is increasing [73]. Other approaches include the implementation of standardized patient treatment pathways, the use of the most modern technological innovations, coordination with other health care providers, the planned introduction of nationwide telephone health advice, and a psychiatric emergency service [6]. The lack or absence of corporate support can lead to a risk of losing employees to competition. The performance of demotivated employees usually decreases, and the company employs low performers. A lack of productivity, lack of motivation, and workplace-related illnesses are often the result [6].

Finally, however, society is also called upon: we must learn to recognize the limitations of emergency medicine. Ambulance services should not be misused. Boundless rescue or “choosing wisely” with the aim to reduce unnecessary medical services are frequent points of discussion [76]. Strategies to reduce the number of emergency transports for noncritical patients, i.e., dispatching an alternative nonmedical transport or mobile care team or deciding not to transport a patient, need to be developed and implemented using standardized queries [76].

### 4.2. Limitations

Although rated low, the studies showed a landmark trend. This trend cannot be explained away despite the low level of evidence. It should the difficulties to draw final conclusions about causal effects on job satisfaction and work engagement in view of the many cross-sectional studies and only a single longitudinal study in the review. The studies did not consider emergency physicians, although there is a high need for research in this area as well. Emergency physicians also have a high burnout rate [18,19]. It is questionable whether the professional titles and the corresponding qualification are the same in all countries. For example, in Germany, there are three different levels of emergency service medical personnel [42]. In other countries, there is no special training for EMSP [37]. Nevertheless, these studies were included because the work could be clearly classified as part of the prehospital setting. One study demonstrated that higher qualification was a predictor of higher job satisfaction and that lower qualification was a negative predictor [24]. Because of the standardization of the parameters collected, a beta weight only indicates the strength of the relationship relative to the distributions of the variables. This could result in a bias due to a sampling error; for example, not surveying the whole population, only a part, and then taking the mean and standard deviation of this small sample to standardize the variables. However, this is a common procedure. The review included studies of younger or middle-aged employees of ambulance services. Transferability to older workers is limited. Older people also tend to have more work experience, which was a positive predictor in this review [24,37]. Only a quarter of the subjects were females, so again, there is a limited possibility of evaluation for women in ambulance services.

## 5. Conclusions

The ambulance service is changing. Corporate support, e.g., supervisors’ support and operational health management, is a key part of job satisfaction and work engagement for EMSP. For this purpose, there is a need for research which, in addition to organizational measures, is primarily concerned with intervention measures for health promotion and management in the workplace. Stress management and resilience should be strengthened. It is also useful to measure not only subjective stress but also objective stress.

- Better pay, benefits, and career advancement opportunities are necessary.
- Emergency medical services should not be a profit-maximizing, competitive service. The health of all parties involved (patients and EMSP) is the primary concern. Emergency rescue stations and personnel deployments must be possible even without high utilization.
- Education is needed for hospitals and doctors’ offices about the types of transport (e.g., ambulance, emergency ambulance) that will be alerted and when.
- The collaboration of hospitals and ambulances services should be improved and requires a deep understanding of the roles of the partners. Ambulance service and disaster management benefit when hospitals provide physicians for missions. This applies to physicians with emergency qualifications.

## Figures and Tables

**Table 1:** Search strategy of the review.

	Terms
	(ambulances[MeSH Terms]) OR (air ambulances[MeSH Terms]) OR (emergency medical service[MeSH Terms]) OR (Emergency Medical Technicians[MeSH Terms]) OR (rescue work[MeSH Terms]) OR (frontline)
AND	(job satisfaction[MeSH Terms]) OR (reward[MeSH Terms]) OR (pleasure[MeSH Terms]) OR (strain) OR (working conditions) OR (subjective importance work) OR (willingness) OR (stress, psychological[MeSH Terms]) OR (occupational stress[MeSH Terms]) OR (occupational stress)
AND	(work engagement[MeSH Terms]) OR (work-related ambition) OR (willingness work exhausted) OR (effort-reward imbalance) OR (demand-control model) OR (commitment)
NOT	(nurses[MeSH Terms]) OR (nursing[MeSH Terms])
NOT	(quality interview)

**Table 2:** Inclusion and exclusion criteria of the review.

	Terms
Inclusion criteria	Quantitative observational studies published in peer-reviewed journals Full text in English or German language Subjects: employees of emergency medical service (EMS) or emergency physicians (EP) More than 20 participants for group comparisons All psychosocial work factors and job characteristics derived from EMS self-reports or expert observations All job satisfaction outcomes derived from individual EMS self-reports or expert evaluations depending on effort–reward imbalance or demand control model All work engagement factors derived from EMS self-reports or expert observations Longitudinal intervention programs with presentation of outcome Personnel working in hospital setting or first responders only as control group to EMS or emergency physicians From 2000 to date of literature search Humans
Exclusion criteria	Personnel only working in hospital settings such as emergency department First responders without completed medical training Other study types, single-case studies, review articles, short communications, letters with insufficient information to analyze the results, guidelines, theses, dissertations, qualitative studies, scientific conference abstracts, studies on animals, and experimental studies

**Table 3:** Associations and predictors for job satisfaction in studies that used job satisfaction questionnaires.

First Author, Year, Country	Characteristics of Subject	Job Satisfaction	
	n, Sex, Age	Coefficient	Correlation Analysis and/or Regression Analysis
**Basabr, 2018** Iran	232, 100% male; 31–40 years	R², ß	***Regression analysis:*** **ß:** gender (−0.202) **, educational level (0.233) ***, monthly income (0.224) ***, work experience (0.238) ***, R² = 0.201 ***
Brough, 2005 Australia	119, 79% male; 28–37 years	r, R², ß	***Correlation:*** **r:** verbal violence (−0.31) ***, psychological strain (−0.25) **, supervisor support (0.44) ***, colleague support (0.33) *** ***Hierarchical multiple regression:*** **ß:** verbal violence (−0.21 **), supervisor support (0.28 **), colleagues support (0.21 **), R² = 0.26 ***
**Carriére, 2008** Canadian	91 74.9% male, 32.8 ± 8.3	r, R², ß	***Correlation:*** **r:** communication satisfaction (0.72) **, affective organizational commitment (0.73) **, communication practices (0.48) **, shift pattern (0.30) **, organizational tenure ((−0.32) ** ***Linear regression analysis:*** * ß: organizational tenure (−0.31) *, shift pattern (0.43) **, R² = 28.4%. **ß:** communication practices (0.47) ***, R² = 19.8%, **ß:** communication satisfaction (0.66) ***, R² = 37%
**Eiche, 2021** Germany	2500, 80.4% male, 18–70 years	OR	***Binary logistic regression*** **OR:** “Notfallsanitäter in training” (OR 3.085) *, “Rettungsassistent” (OR 0.691) **, “0–5 years of service” (OR 1.588) **, “age 18–30 years” (OR 1.977) **, “age 21–40 years” (OR 2.263) **
**Stefurak, 2020** USA		R, η²	***Correlations:*** **r:** public service motivation (0.430) **, compassion (0.227) ** Multivariate analysis of variance: **η²:** public service motivation (0.155)
**Sterud, 2011** Norway	324, 76.8% male, 18–66 years	r, R², ß	***Correlations at t2:*** **r:** emotional exhaustion at t2 (0.500) *, psychological distress at t2 (0.300) *, musculoskeletal pain at t2 (0.140) *, lack of co-worker support (0.240) * and severity level (0.200) *, lack of leader support frequency level (0.360) * and severity level (0.340) *, time pressure frequency level (0.150) * and severity level (0.180) *, challenging job task severity level (0.300) *, physical demand frequency level (0.12) * and severity level (0.200) * Multiple regression **ß:** extroversion (0.12) *, lack of leader support frequency level (0.32) ***, challenging job task severity level (0.27) **

Notes: ß = Standardized beta-weighted regression coefficient, Significance level: * *p* < 0.05, ** *p* < 0.01, *** *p* < 0.001. Interpretation of the Pearson’s or Spearman’s correlation coefficients: <0.39 low, 0.40–0.59 moderate, >0.6 strong effect.

**Table 4:** Associations and predictors for job satisfaction in studies that used job satisfaction as items in other questionnaires.

First Author, Year, Country	Characteristics of Subject	Job Satisfaction	
	n, Sex, Age	Coefficient	Correlation Analysis and/or Regression Analysis
**Nowrouzi-Kia, 2022** Canada	879, 71.6% male, 38.1 ± 11.85	R², ß	***Multiple Linear Regression:*** **ß:** gender (0.245) *, R² = 0.145 **
**Roth, 2021** Germany	1082, 86.1% male, ≤ 29- ≥ 50 years,	r, R², ß	***Correlation* (variables job satisfaction with emotional exhaustion and depersonalization):** **r:** current workplace (−0.373/−0.140) **, career choice (−0.230/−0.146) **, flexibility of shift schedule (−0.228/−0.112) **, autonomy at work (−0.130/−0.134) **, professional status (−0.150/−0.140) **, salary (−0.140/−0.121) **, vacation days (−0.170/−0.093) **, disease regulation (−0.204/−0.087) **, training days (−0.195/−0.077) */** Regression analysis (emotional exhaustion/depersonalization if significant): **OR:** current workplace (0.270) ***, career choice (0.574/0.696) **/*, professional status (0.638/0.618) **

Notes: ß = Standardized beta-weighted regression coefficient, Significance level: * *p* < 0.05, ** *p* < 0.01, *** *p* < 0.001. Interpretation of the Pearson’s or Spearman’s correlation coefficients: <0.39 low, 0.40–0.59 moderate, >0.6 strong effect.

**Table 5:** Associations and predictors for work engagement or commitment.

First Author, Year, Country	Characteristics of Subject	Job Satisfaction	
	n, Sex, Age	Coefficient	Correlation Analysis and/or Regression Analysis
**Naudé, 2006** South Africa	323, 80% male, 33.13 ± 8.08	r, R², ß	***Correlation:*** **r:** emotional exhaustion (−0.280) **, depersonalization (−0.320) **, personal accomplishment (0.610) **, sense of coherence (0.41) ** ***Multiple regression analyses*** **ß:** sense of coherence (0.160) **, R² = 0.18
**Setti, 2018** Italian	353, n.r. 41.9 ± 15.3	r ß	**Correlation:** **r:** supervisors support (0.280) **, colleagues support (0.230) ** Model for Testing Mediation Effect: role conflict (β = 0.16), supervisors support (β = 0.25), job burnout (β = −0.22)

Notes: ß = Standardized beta-weighted regression coefficient, Significance level: ** *p* < 0.01. Interpretation of the Pearson’s or Spearman’s correlation coefficients: <0.39 low, 0.40–0.59 moderate, >0.6 strong effect.

## Data Availability

The data can be requested from the authors.
